# The Rapeseed Potential in Poland and Germany in the Context of Production, Legislation, and Intellectual Property Rights

**DOI:** 10.3389/fpls.2019.01423

**Published:** 2019-11-05

**Authors:** Ewa Woźniak, Ewa Waszkowska, Tomasz Zimny, Sławomir Sowa, Tomasz Twardowski

**Affiliations:** ^1^Institute of Bioorganic Chemistry, Polish Academy of Sciences, Poznań, Poland; ^2^Examining Department, The Patent Office of the Republic of Poland, Warsaw, Poland; ^3^Institute of Law Studies, Polish Academy of Sciences, Warsaw, Poland; ^4^Department of Plant Breeding, Swedish University of Agricultural Sciences, Alnarp, Sweden; ^5^Plant Breeding and Acclimatization Institute (IHAR)—National Research Institute, Radzików, Poland

**Keywords:** agricultural innovation, new breeding techniques, patents, plant variety rights, rapeseed

## Abstract

Rapeseed is an essential crop which is used in many different areas as edible oil, biodiesel, lubricant, and feed. It is one of the most popular oil crops in Europe (63% of oilseeds production in 2017). The current study highlights the potential for further rapeseed development in European Union (EU), with special emphasis on Germany (19% of EU production) and Poland (12% of EU production). The study focused on three factors: cultivation area, volume of production and the numbers of Intellectual Property Rights (IPR), particularly patents granted for rapeseed or rapeseed-related inventions and plant variety rights. Possible further obstacles to development, such as current legal framework, were also taken under consideration. The analyzed statistical data shows that both the cultivation area, as well as the volume of production of rapeseed fluctuated in the last decade in both examined countries, while the numbers for European patent publications and Community Plant Variety Rights showed a rising trend, indicating investments in the Research and Development (R&D) of the crop. The data analysis seems to confirm a hypothesis that there is a potential for the development of rapeseed as a versatile, multi-use crop; however, the current EU GMO policies and a legal uncertainty as to the status of products of certain modern gene editing techniques may hamper making optimal use of this potential.

## Introduction

Rapeseed has multiple applications viz. human food, cattle feed, and for industrial purposes as a source of biodiesel or bioethanol. Research is being carried out to utilize not only seeds and oil cake (Negahdar et al., 2016; Kdidi et al., 2019) but also other by-products of oil production, such as straw (Wang et al., 2019). Rapeseed production may improve the sustainability of land use, which may also require advancements in the genetic diversity of the plants and hence the development in breeding itself, to achieve efficient use of genetic resources through biological progress. However, certain legal obstacles, in particular after the recent Court of Justice of the EU judgement in the C-528/16 “mutagenesis” case, which seems to limit the choice of breeding methods available to EU breeders (see sec. 3.3), may hamper this developmental potential and put EU breeders at a disadvantage in comparison to their competitors from other countries.

According to the Food and Agriculture Organization of the United Nations in 2017, the world production of oilseeds (rapeseed, sunflower seed, soybean, linseed) amounted to 479 million tons, whereas consumption amounted to 492 million tons (FAOSTAT, 2018; OECD-FAO, 2018). Soybean had the largest share of oilseed production in the world in 2017 (73%), whereas rapeseed was classified in second place, with a share of 16%.

The European Union (EU) was the world leader in rapeseed production in 2017 (22 million tons). The next places were occupied by Canada (21 million tons), China (13 million tons), India (7.9 million tons), Australia (4.3 million tons), and Ukraine (2.1 million tons) (FAOSTAT, 2018). According to the statistics of the Institute of Agricultural and Food Economics - National Research Institute in Poland (2018), the greatest producers of rapeseed in the EU are the following: France, Germany, Poland, Romania, Great Britain, the Czech Republic, Hungary, Denmark, and Slovakia.

There are different common names of the *Brassica napus* that are well-known for high oil content: rapeseed, rape, canola. The name “canola” is derived from the words “Canada” and “oleo” (oil) and is used to describe rapeseed varieties with low erucic acid and low glucosinolate content in extracted edible oil (Canola Council, 2017). Rapeseed oil is commonly used for cooking, lighting, industrial uses, and feed, especially rapeseed meal and rapeseed cake, which are by-products of oil production. Rapeseed meal contains approximately 36–38% protein and 2–4% fat (Brzóska et al., 2010). Rapeseed cakes are moister than rapeseed meal and contain 10–14% fat. Moreover, rapeseed feeds contain more mineral ingredients than soybean meal (calcium, iron, manganese, phosphorus, magnesium, and selenium) (Woźniak and Twardowski, 2018).

Rapeseed meal is used as valuable feed; however, there are strict limitations concerning the amount used in animal feeding, due to its specific properties (Herkes, 2019). Rapeseed contains glucosinolates, which are the main antinutritional factor that hinders the animal nutrition by making chelates with minerals, which cause unavailability of essential minerals to animals during digestion (Kaczmarek et al., 2016). There is a chance of increasing the share of feed components derived from rapeseed. However, the high fiber content (up to 16%) that affects the digestibility of animals must be highlighted. The fiber content of the seeds and by-products of rapeseed can be reduced through breeding and development of new varieties (Ogrodowczyk and Bartkowiak-Broda, 2013). Canola and rapeseed contain approximately 40% oil. Canola oil is high in oleic acid, which makes it competitive with other cooking oils. Moreover, the oil is also a high-grade lubricant and fuel additive; therefore, conversion to biodiesel is just one of its several potential final uses (Herkes, 2019).

An enterprise can strengthen its market position and gain a competitive advantage through utilization of Intellectual Property Rights (IPR). Such exclusive rights provide a means for obtaining a return on investment in research and development (R&D), through licenses or the transfer of rights. IPR facilitate technology transfers and allow for access to new markets. The monopoly granted by exclusive rights provides owners with an ability to efficiently protect themselves against infringers (Andrews and Criscuolo, 2013). Hence, various forms of IPR are typically obtained by entrepreneurs working in innovative fields. An analysis of numbers of intellectual property rights granted can provide information on R&D investments in a given area of technology (Griliches, 1998).

One of the indicators of development of various industries, including agriculture, is the amount of granted and commercialized patents and the rate of change of this amount. A patent grants an exclusive right to commercially use, distribute, and license the protected invention. The granting of patents, licenses, and other proprietary rights facilitates and triggers company’s development; allows it to enter a higher, global level of operation; and can also become an important part of the entity’s revenues (European Commission, 2013). A broad patent portfolio may also contribute to the company’s market value (Coad and Rao, 2006). Patents are granted by specialized authorities, such as the European Patent Office (EPO) or national patent offices and are listed in publicly available databases, making them relatively easy to research and assess statistically.

In the field of plant breeding, there is also an alternative exclusive right in use, namely the plant variety right, which provides its owner with exclusivity when it comes to commercialization of their variety. Like patents, plant variety rights are granted by a specialized office (e.g., the Community Plant Variety Office—CPVO—in the EU) and are collected in publicly available databases.

The aim of the study was to analyze the potential for development of rapeseed in Europe, with an emphasis on Poland and Germany. The tested hypothesis was that there is indeed a potential for the development of rapeseed as a versatile, multi-use crop. Three indicators were used in the analysis: the overall cultivation area and its changes over time, the volume of production and its changes over time, and the numbers of IPR (patent publications concerning rapeseed and plant variety rights granted) and the changes of those numbers over the years. The choice of those indicators allows to see not only the utilization of the crop but also the dynamics of R&D investments in technologies surrounding it. Additional potentially limiting factors, such as legal obstacles, were also taken into account. Poland and Germany were singled out due to the similarities of their markets, comparable climatic conditions, their mutual co-dependency and strong commercial relations, and due to the fact that they account for over 30% of overall rapeseed production in the EU.

It was not the aim of this study to present IPR as means for increasing the rapeseed potential. The data on patent and plant variety rights were merely used as indicators of the condition and prospects for rapeseed development, alongside other factors, such as cultivation area and legal obstacles.

## Materials and Methods

The study used research data published by Directorate-General for Agriculture and Rural Development (DG AGRI, 2019) and Eurostat as well as data from the Central Statistical Office (CSO) of Poland in order to gather data about the land use and production volume of rapeseed and their changes in time.

Patent data can be used as indicators of technological development of multiple areas of technology, including biotechnology (Pilkington et al., 2002; OECD, 2005; OECD, 2008; Dubarić et al., 2011). In particular, there is a strong correlation between R&D investments and the number of patents (Griliches, 1998). In order to determine the developmental prospects in the field of biotechnology of rapeseed, the databases of the German Patent Office (DEPATISnet), the Polish Patent Office (PPO), and the European Patent Office (EPO—Espacenet) were examined in this study. The numbers of patent publications regarding rapeseed year by year were examined and compared. The data relating to patents were collected based on International Patent Classification (IPC, 2019.01 version)^1^ codes and keywords.

Following the guidelines outlined in OECD (2005, 2008), a search encompassing the years between 1999 and 2017 was performed within the abovementioned databases. A presence of specific classification codes in a patent application (indicated by examiners of patent offices) means an affiliation with a specific industrial sector. **Table 1** shows the IPC classes, in which the number of inventions involving oilseed rape is represented most frequently. A full text search (i.e. including the title, abstracts, description and patent claims) was performed for each year of the date range. IPC classes typical for the area of biotechnology (OECD, 2008) were included in the results. Consequently, classes outside the area of biotechnology (e.g. machinery) were excluded from the search. To properly understand the meaning of the IPC codes indicated in a patent, it is necessary to know that one invention usually has several IPC codes, e.g., invention—a method of producing fat for chocolate products has an IPC code for the C07 class—organic chemistry and A23—food, in general.

**Table 1:** Definitions and contents of the most relevant IPC classes.

IPC symbol	CONTENT
A01	agriculture, forestry, animal husbandry, hunting, trapping, fishing – soil working in agriculture or forestry – planting; sowing; fertilizing – harvesting – horticulture; cultivation of vegetables, flowers, rice, fruit, vines, hops, or seaweed; forestry; watering – new plants or processes for obtaining them; plant reproduction by tissue culture techniques – manufacture of dairy products – preservation of bodies of humans or animals or plants or parts thereof – biocides, e.g., as disinfectants, pesticides or herbicides – biocidal, pest repellant, pest attractant or plant growth regulatory activity of chemical compounds or preparations
A21	baking, for making or processing doughs, doughs for baking – handling baked articles made from dough – treatment, e.g., preservation of flour or dough for baking, e.g. by addition of materials; baking; bakery products; preservation thereof
A23	foods or foodstuffs – preserving, e.g., by canning, meat, fish, eggs, fruit, vegetables, edible seeds; chemical ripening of fruit or vegetables; the preserved, ripened, or canned products – dairy products, e.g., milk, butter, cheese; milk or cheese substitutes; making thereof – edible oils or fats, e.g., margarines, shortenings, cooking oils – coffee; tea; their substitutes; manufacture, preparation, or infusion thereof – protein compositions for foodstuffs; working-up proteins for foodstuffs – feeding stuffs specially adapted for animals; methods specially adapted for production thereof – foods, foodstuffs, or non-alcoholic beverages – preservation of foods or foodstuffs
A61	medical or veterinary science; hygiene – preparations for medical, dental, or toilet purposes – specific therapeutic activity of chemical compounds or medicinal preparations
C07	organic chemistry – general methods of organic chemistry – organic compounds
C08	organic macromolecular compounds; their preparation or chemical working-up; compositions based thereon
C09	dyes, paints; polishes; natural resins; adhesives; compositions not otherwise provided for; applications of materials not otherwise provided for
C10	petroleum, gas or coke industries; technical gases containing carbon monoxide; fuels; lubricants; peat
C11	animal or vegetable oils, fats, fatty substances or waxes; fatty acids therefrom; detergents; candles
C12	biochemistry, beer; spirits; wine; vinegar; microbiology; enzymology; mutation or genetic engineering

Source: based on WIPO classification (2019).

The numbers of inventions involving rapeseed plants or their products were identified for each year. The term “involving rapeseed” means inventions concerning the plant itself, as well as products thereof, used for achieving inventions in any process. Therefore, a broad spectrum of rapeseed applications was included in the research study. The patent data analysis does not distinguish between domestic and foreign applicants in a given patent office. For instance, the numbers obtained for the PPO include both Polish and foreign applicants. The term “Polish application” means an application filed with the PPO, “German application” means an application filed with the German Patent Office, etc. The study could render more precise results as to the specific uses of patented inventions, but this would require a detailed analysis of the contents of the thousands of identified patent documents and could not be carried out within the ramifications of this project.

Obtained data were gathered for each of the patent offices separately and plotted on the charts (**Figures 3**–**8**). Trends indicating prospects for development for each of the offices were calculated using least squares regression analysis carried out in the Statistica software.

The sample of plant variety rights analyzed in this paper was derived from the Community Plant Variety Rights Office (CPVO) database. The search encompasses plant variety rights granted for rapeseed between 1995 (the creation of the database) and 2018 (last full year of the study). Initially, 788 plant variety rights for rapeseed were identified within the examined period. Those were consequently matched with the year in which they were granted.

Standard least squares regression analysis was carried out for the gathered data set in order to plot the trend line within the analyzed period. The sample was also filtered by the nationality of subjects to whom rights were granted, in order to see the scope of innovative activity and the willingness to protect its effects in Poland and Germany, respectively. A company was treated as a company based in a particular country if its main seat is based in said country. Hence, the number of rights granted to, e.g., a German company, includes those granted to its subsidiaries in other countries. Conversely, rights granted to a German branch of a company based in a different country were rejected.

## Results and Discussion

### Economic Aspects of Rapeseed Cultivation in the EU

**Figure 1:**
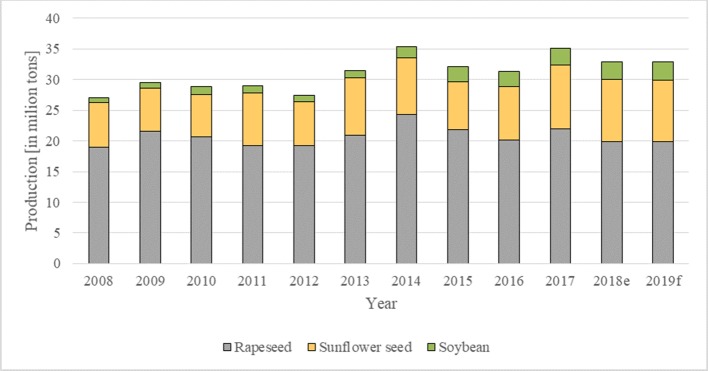
Production of oilseeds in EU 2008–2019, (f – forecasted production; e – estimated production). Source: own study based on data from DG AGRI and Eurostat 2019.

According to the data from DG AGRI in the EU in 2017, the production of oilseeds (rapeseed, sunflower seed, soybean, linseed) amounted to 35 million tons, with rapeseed accounting for 22 million tons (63% of oilseeds). EU was the largest rapeseed producer (approximately 30% of world production) (FAOSTAT, 2018). The highest rapeseed production, 24 million tons, was in 2014 (see **Figure 1**).

The forecasted production (f) of oilseeds in 2019 is lower by approximately 2.2 million tons than in 2017 (**Figure 1**). However, according to the data (DG AGRI, 2019) the production of rapeseed is anticipated to increase by 18 000 tons in 2019.

**Figure 2:**
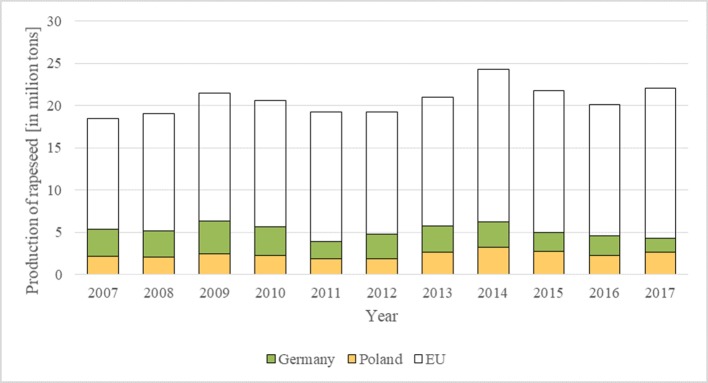
Production of rapeseed in Germany and Poland from 2008 to 2017. Source: own study based on data from DG AGRI and Eurostat 2019.

The production of rapeseed in Poland in 2017 amounted to 2.7 million tons, whereas production in Germany amounted to 4.3 million tons (**Figure 2**). The volume of rapeseed production changed significantly from 2008 to 2017. The production of rapeseed in Germany is higher than in Poland; however, differences between the countries are decreasing year by year. Rapeseed can be the fastest developing crop in Poland, mainly because of its use in biodiesel production. However, data for the last decade show a slight decrease in the use of rapeseed as a source of biodiesel (USDA EU-28, 2019) concurrently with an increase in the overall use of biodiesel. Biodiesel is the most important among many types of biofuels produced and used in the EU (Sorda et al., 2010). Spain, Germany, and France lead among EU countries in biodiesel production (Eurostat, 2019). According to Eurostat (2019), in 2016, biodiesel production in EU countries reached approximately 21 million tons. In Poland, the production of biodiesels accounted for 1.15 million tons, whereas in Germany, it was 4.1 million tons.

In 2017, in EU countries, the area of rapeseed accounted for 6.7 million hectares (**Table 2**). In Poland, it was 0.91 million hectares (14% of the area of rapeseed in EU), whereas, in Germany, it was 1.3 million hectares (19.4% of the area). Due to dry sowing conditions in some major rapeseed producing countries, rapeseed acreage has declined sharply, especially in France and Germany (USDA EU-28, 2019). The area of rapeseed cultivation in Poland and Germany was characterized by frequent changes resulting from decrease of planting and unfavorable weather conditions. In Poland, a 19% increase in cultivated area was observed in 2017 compared to 2008. In Germany in 2017, the area of cultivation for this plant was decreased. Nevertheless, when looking from a longer time perspective, one can observe a steady upward trend in Poland since the 1950s combined with a sudden increase in the decade between 2005 and 2015 (from 0.55 million to 0.95 million hectares) and a plateau in recent years (CSO, 2018). This finding may indicate that, for now, the demand for rapeseed has stabilized.

**Table 2:** The area of rapeseed cultivation in the EU, Germany, and Poland in millions of hectares.

	2008	2009	2010	2011	2012	2013	2014	2015	2016	2017
**EU-28**	6.1736	6.5307	7.1056	6.7483	6.2091	6.7136	6.7144	6.4672	6.5347	6.7488
Germany	1.3707	1.4712	1.4612	1.3286	1.3062	1.4656	1.3942	1.2855	1.3257	1.3089
Poland	0.7711	0.8100	0.9461	0.8301	0.7203	0.9207	0.9511	0.9471	0.8226	0.9143

Source: own study based on data from DG AGRI and Eurostat 2019.

EU imported 3.6 million tons of rapeseed in 2013–2017, mainly from Australia (44%) and Ukraine (36%). It is worth to mention that in 2018–2019, import of rapeseed increased to 4.2 million tons (EU Oilseed Complex, 2019). A significant share of the EU oilseed plants import belongs to GM soybean. According to the EU Commission, between 2014 and 2016, the EU imported more than 30 million tons of GM soybean annually, including Poland, which imported 2 million tons (Rostoks et al., 2019). Replacing imported GM soybean meal with domestically grown oilseed, such as rapeseed, in the feed industry is currently not an achievable and practical alternative because of technical and climatic limitations.

### Patents and Plant Variety Rights—Analysis and Comparisons

#### European Patents

**Figure 3:**
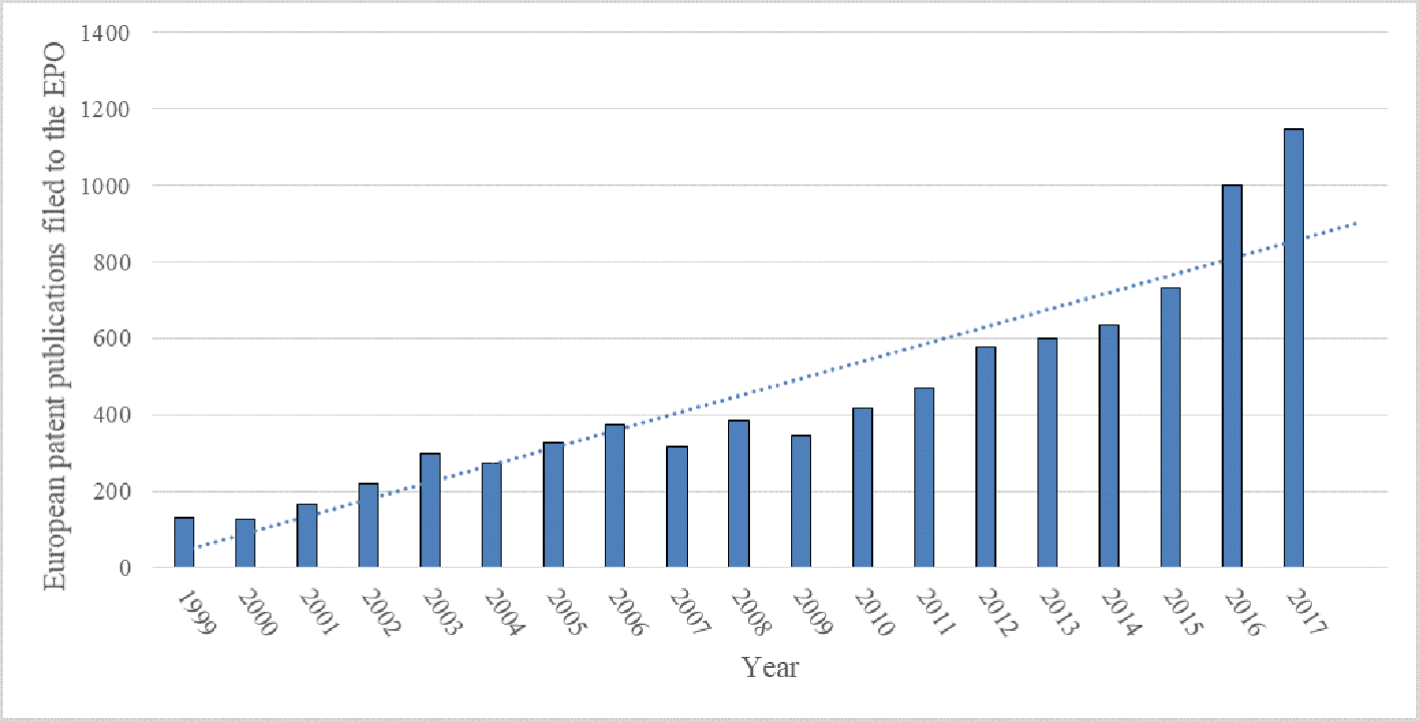
Number of European patent publications filed to the EPO from 1999 to 2017 that use rapeseed or any product thereof and their trend (full text search). Source: own study based on data from Espacenet.

**Figure 4:**
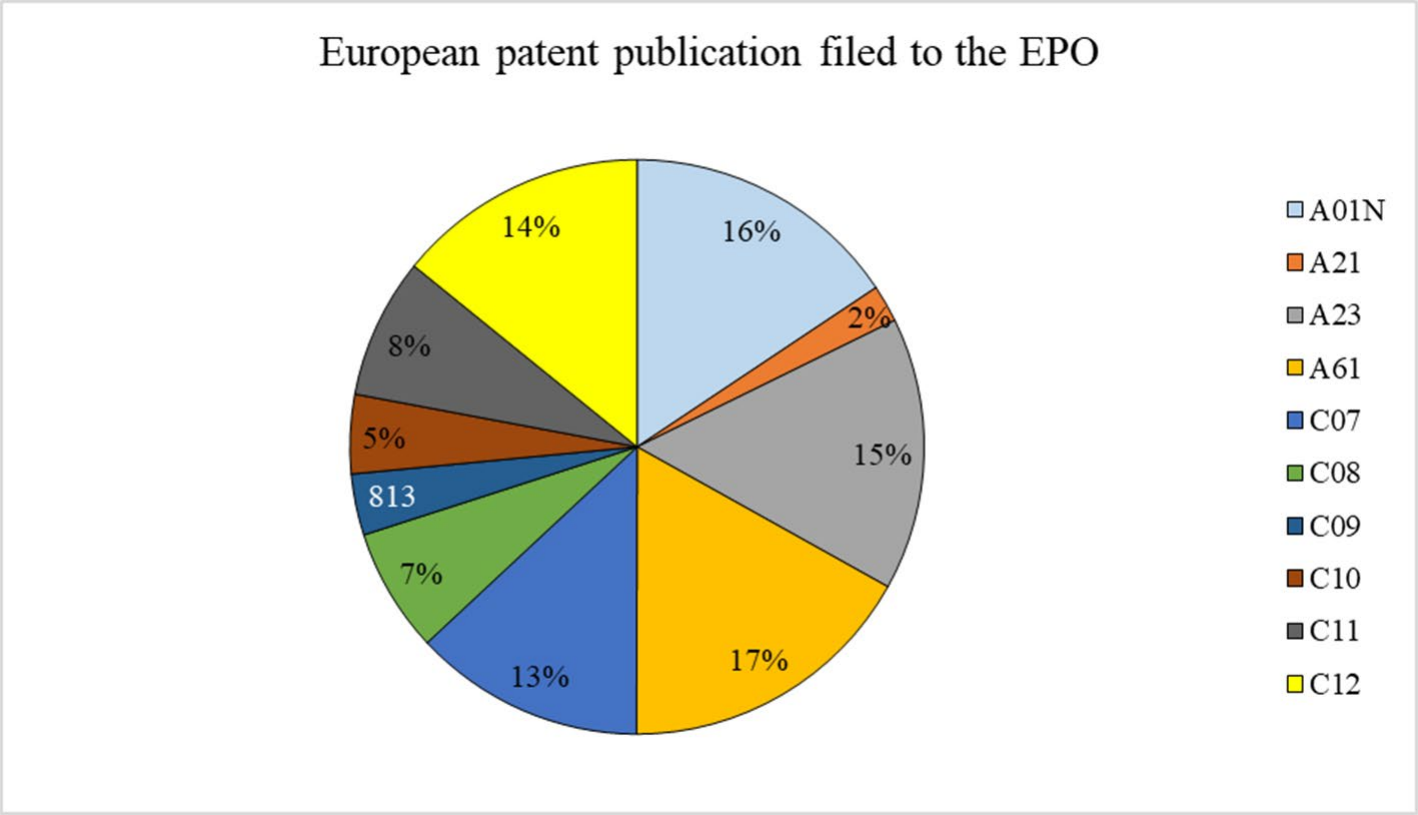
European patent publications (in %) filed to the EPO from 1999 to 2017 that use rapeseed itself or any product thereof in different industrial fields (full text search). See **Table 1** for the explanation of the symbols. Source: own study based on data from Espacenet.

The number of European patent publications was characterized by a steady increase between 1999 and 2017 (**Figure 3**). A look at different IPC classes, where none are dominating (**Figure 4**), shows a widespread research and use of rapeseed in different areas and may be seen as reflecting its industrial potential in agriculture, i.e., soil working, planting, harvesting, cultivating vegetables, developing new plants or the processes for obtaining them, and manufacturing dairy products, as well as in biocides, e.g., disinfectants, pesticides or herbicides, or plant growth regulators.

The numbers of patent publications per year are an order or in some years two orders of magnitude higher than for either of the national offices examined. This does not necessarily indicate the level of R&D investments in respective countries, but rather the popularity of each of the examined offices. A European patent can provide protection in multiple European countries, including Germany and Poland. Unlike in the case of the national offices, what stands out in the presented chart (**Figure 3**) is a continual growth of the number of patent publications each year. This in turn, coupled with the abovementioned correlation between patent data and the R&D investment levels can indicate R&D investments in rapeseed and rapeseed connected technologies. Since patents are granted for inventions that have to show improvements in comparison to previously patented ones, a growing trend indicates an acceleration of development of a given area year to year (even a horizontal trend line in this case would mean that new solutions are being developed each year and as such would show progress, not stagnation).

#### Polish Patents

**Figure 5:**
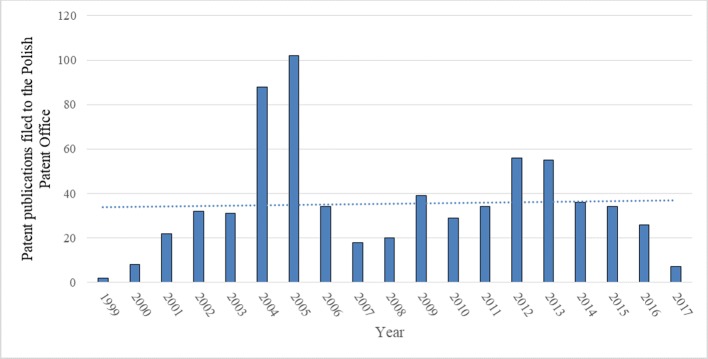
Number of patent publications filed to the Polish Patent Office from 1999 to 2017 regarding inventions that use rapeseed or any product thereof, and their trend (full text search). Source: own study based on data from the Polish Patent Office (2018).

**Figure 6:**
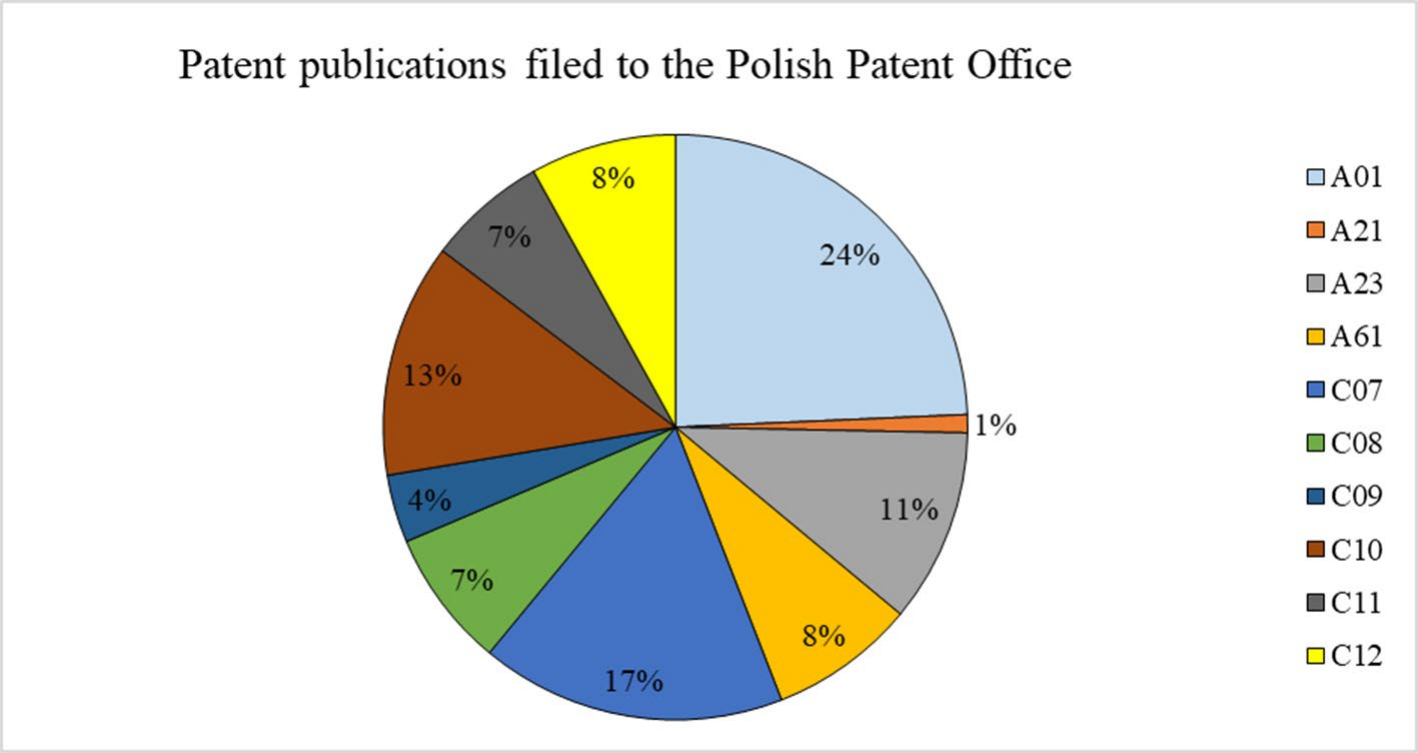
Polish patents (in %) filed to the Polish Patent Office from 1999 to 2017 that use rapeseed itself or any product thereof in different industrial fields (full text search). See **Table 1** for the explanation of the symbols. Source: own study based on data from the Polish Patent Office (2018).

The data on Polish patents show a frequent year to year changes with almost a horizontal trend line (**Figure 5**). The highest number of patents granted in the PPO are applications in the IPC class A01—24% (**Figure 6**). It was to be expected, as this is the broadest class covering agriculture, forestry, animal husbandry, hunting, trapping, and fishing. Organic chemistry (class C07) is the second most common application—17% (**Figure 6**). It is important to note that, in Poland, many inventions (13%) cover the use of rapeseed in the petroleum or gas industries (IPC-C10).The number of Polish patents for genetically modified (GM) rapeseed itself or involving any products thereof in genetic engineering was 22 from 1999 to 2017 (C12N15/82 of IPC).

#### German Patents

**Figure 7:**
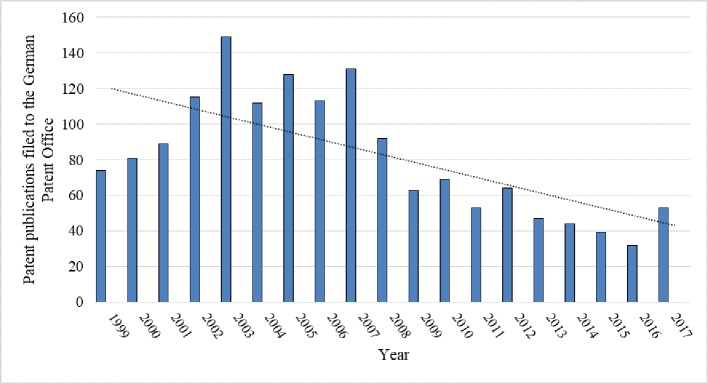
Number of patent publications filed to the German Patent Office from 1999 to 2017 regarding inventions that use rapeseed or any product thereof and their trend (full text search). Source: own study based on data from DEPATISnet (2019).

The number of patent publications changed in the period from 1999 to 2006 and remained at a high level (**Figure 7**). The number of patent publications in Germany was characterized by frequent changes. After 2007, the number of patent publications of the German Patent Office decreased. There are clearly marked years of stable growth (1999–2007) and years of decline (2008–2017). This observation should be analyzed in detail in the future.

**Figure 8:**
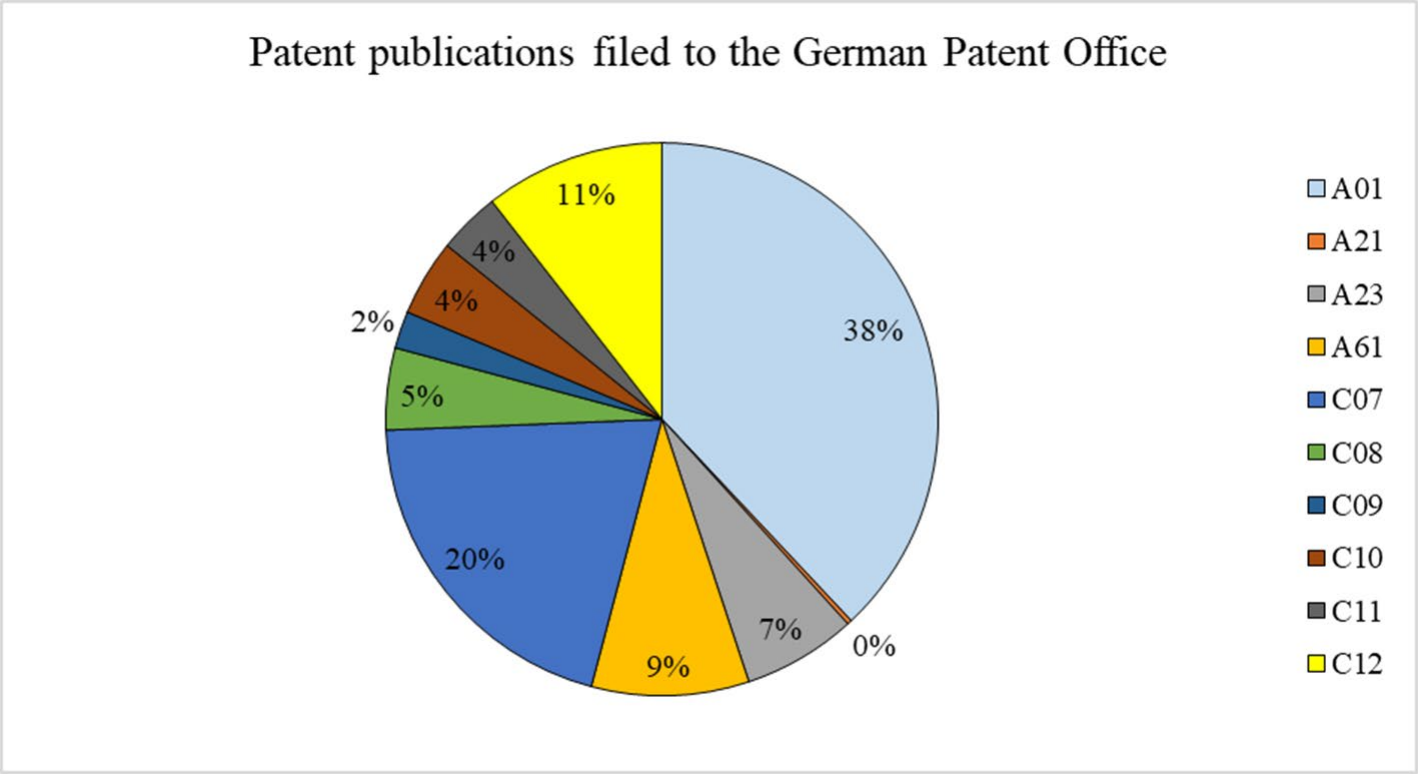
Patent publications (in %) filed to the German Patent Office from 1999 to 2017 that use rapeseed itself or any product thereof in different industrial fields (full text search). See **Table 1** for the explanation of the symbols. Source: own study based on data from DEPATISnet (2019).

The largest proportion of patent publications filed in the German Patent Office came from agriculture (38%), organic chemistry (20%), and biochemistry (11%) (**Figure 8**). The number of German patent applications regarding GM rapeseed itself or involving any products thereof in genetic engineering was 186 between 1999 and 2017 (C12N15/82 of IPC).

#### Plant Variety Rights

Plant variety rights and patents exist simultaneously in Europe, sometimes protecting the same solutions. Although the European Patent Convention (EPC) outright forbids granting of patents for plant varieties (art. 53b), it does not preclude granting patents to groupings broader than a variety, even if such a group encompasses varieties (see EPO, 2018a; EPO, 2018b. Guidelines for Examination to the EPO part G.II.5.4.1). Hence, a patented invention can find its application within multiple protected varieties.

**Figure 9:**
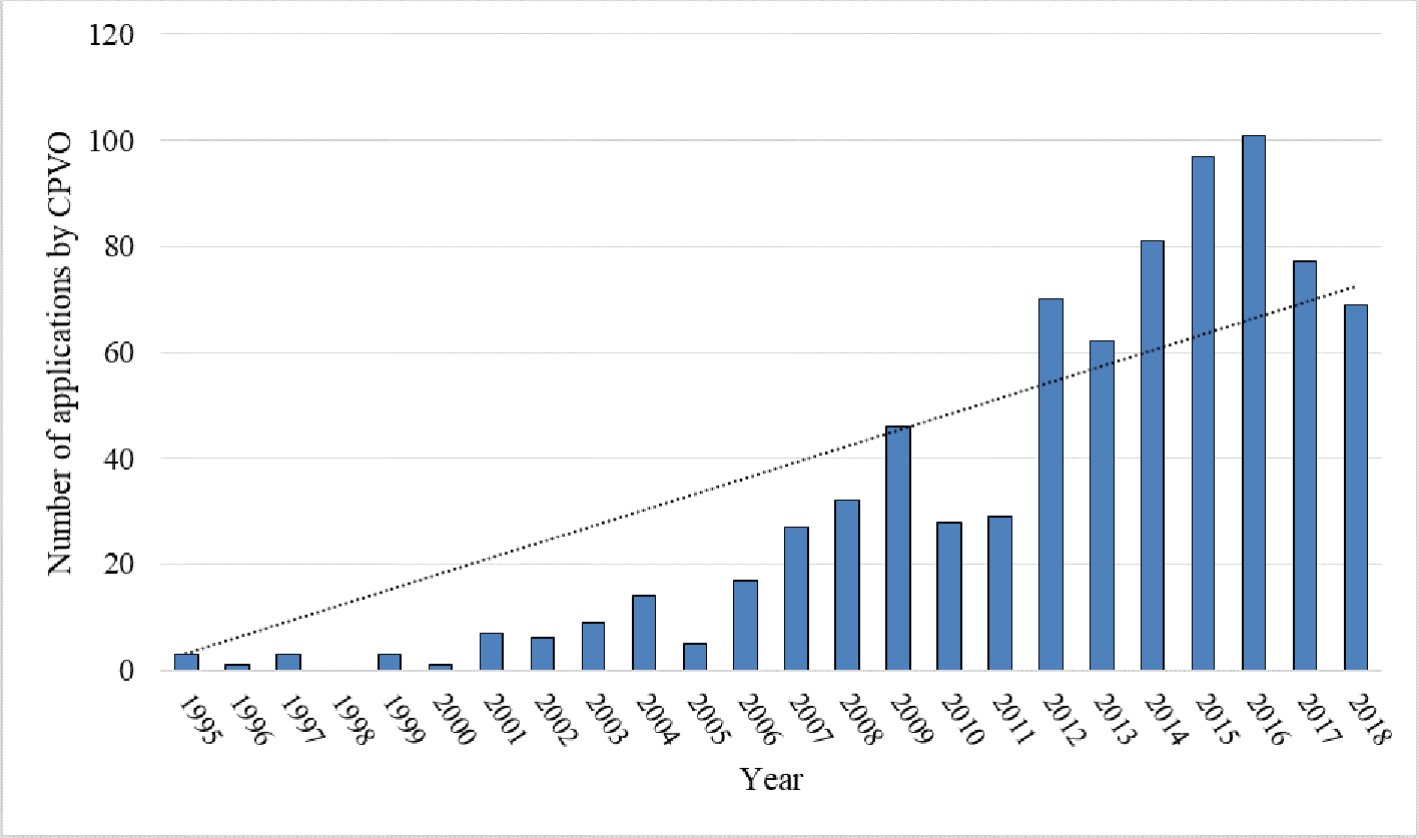
Number of applications for which exclusive plant variety rights were granted 1995–2018 and their trend. Source: own study based on data from CPVO (2019).

Steady progress in the breeding of rapeseed can be observed in the last decade, with the number of plant variety rights granted for new rapeseed varieties growing and clearly exceeding the numbers from before 2010 (**Figure 9**).

In the area of plant variety rights, a domination of large companies can be shown, with seven companies owning over 80% of the rights (see **Table 3**). Out of all the rights granted from 2000 to 2018, over half (396) belonged to German companies and only 2 to Polish companies. The numbers for the years 2017–2018 may not fully represent the number of rights granted, due to a delay in the delivery of decisions. As of May 2019, there were still 6 applications active for 2017 and 32 for 2018. The number of active applications filed already in 2019 was 48.

**Table 3:** Companies to whom plant variety rights were granted.

Company	Number of rights
KWS	191
Monsanto	95
Pioneer	95
Norddeutsche Pflanzenzucht	93
Deutsche Saatveredelung	69
Syngenta	56
Limagrain	41
BASF	29
Euralis	29
Lantmännen	24
RAGT	18
Caussade	16
Saatzucht Donau GmbH & Co. KG	10
JTSD	4
Selgen	4
Knold & Top	3
W. von Borries-Eckendorf GmbH & Co. KG	3
Saatbau Linz eGen	2
Lammers Seed Options	1
Maïsadour	1
Smolice	1
Strzelce	1

Source: based on CPVO (2019)*.*

The data on plant variety rights show that the level of innovativeness in breeding is currently higher than that in the previous decade and, like in the case of European patents, the pace of progress seems to be increasing. Despite rapeseed being a widespread crop in Poland, Polish breeding companies do not seem to apply for EU plant variety rights. This finding may indicate either a low level of innovativeness or the low attractiveness of the rights themselves. In comparison, the number of rights possessed by German companies is higher by two orders of magnitude. Despite having rapeseed production at a comparable level, the Polish farming industry does not seem to contribute to its biological progress as much as the German industry. There may be various reasons for this fact, including the low collectability of royalties in Poland or farmers’ reluctance to grow newer varieties.

### Legal Ramifications of Novel Methods of Breeding in the EU

No GM varieties of rapeseed are grown in the EU, since none were authorized for cultivation. In 2017, the share of GM rapeseed in the global area of biotech crops was 5% (50% of the area was occupied by GM soybean) (ISAAA, 2018). The global area of GM rapeseed increased by 19% from 8.6 million hectares in 2016 to 10.2 million hectares in 2017. This change occurred due to the adoption of new GM rapeseed varieties with nutritious oil content and different types of herbicide tolerant traits. Herbicide tolerant rapeseed is the fifth most important biotech plant trait commercialized since 1996; it has been adopted largely in Canada, the USA, and Australia (ISAAA, 2018). Moreover, the global area and adoption of rapeseed could increase significantly in the near term in response to the likely increased use of rapeseed for vegetable oil and biodiesel (ISAAA, 2018).

The use of GM products as food and feed in the EU is strongly limited due not only to the strictness of criteria but also to the length and uncertainty of the authorization process, which may work as a deterrent when choosing the breeding method (Zimny et al., 2019). A recent dispute regarding the legal status of products of certain new plant breeding techniques (NBTs) from the point of view of EU genetically modified organisms (GMOs) legislation may lead to legal uncertainty and may work as another deterrent when choosing such breeding methods as Site Directed Nucleases or Oligonucleotide Directed Mutagenesis. In a recent judgment in the case C-528/16, the Court of Justice of the European Union ruled that only organisms obtained by “means of techniques/methods of mutagenesis, which have conventionally been used in a number of applications and have a long safety record,” are exempted from the scope of Directive 2001/18/EC on the deliberate release of GMOs to the environment (Directive, 2001). As noted by Smyth and Lassoued (2019), this judgment may have detrimental impacts on agricultural innovations, R&D funding, and international trade.

According to Eriksson et al. (2019a), it is a paradox related to GM food and feed that 62 different transformation events that have passed the risk assessment by the European Food Safety Authority (EFSA) are entering the EU as food and feed, but only one can be planted in the EU (the MON 810 maize). The authors described two scenarios for implementing a national opt-in mechanism for the cultivation of GM plants under EU legislation and highlighted that if member states have the right to opt out of GM crop cultivation, they should also have a right to opt in (Eriksson et al., 2019a).

The legal disputes regarding novel methods of plant breeding, including those applied to rapeseed, may hamper the development of technologies that would allow for the optimal use of rapeseed as a food and industrial crop and its future potential.

## Conclusions

Analyzed data on patent numbers and plant variety rights show potential of rapeseed use in the food and feed industries, as well as in industrial applications (as follows from the analysis of the IPC classes). What clearly results from this work is that rapeseed is a popular crop in both compared countries—Poland and Germany. After a dynamic growth, the acreage of rapeseed cultivation has been rather steady over the last few years and subject to periodical fluctuations. While used mostly as a source of food and feed, rapeseed also has industrial applications. Its use as a substrate for the production of biodiesel has not only stagnated in recent years but also shows a slight decrease.

The continuation of the development of new varieties is required to expand rapeseed cultivation. As indicated previously the EU, Poland and Germany are not self-sufficient in terms of demand for protein feedstuffs and for energy; for this reason, EU imported more than 30 million tons of GM soybean. If imported GM soybean can be replaced by additional oilseeds grown in EU countries, it would require additional area.

Taking advantage of the full potential of rapeseed would require the utilization of whole plants, and such research is already being carried out. Nevertheless, a multi-faceted utilization of rapeseed products requires not only progress in processing technologies but also in breeding (Campbell et al., 2016). It is through breeding that the genetic diversity of rapeseed could be increased so that it could be used as a sustainable product with multiple applications. Countries such as Poland and Germany are particularly suitable for making use of that potential. The comparison of the IPR management policies of those countries’ breeders shows that Germany has a very significant advantage in this respect over Poland. Despite showing comparable demand for rapeseed, both countries differ significantly in regard to the protection of new varieties. It is difficult to explain this phenomenon without analyzing company policies, and such an examination was beyond the scope of the study and may also be hampered by a particular company’s unwillingness to share such policies.

The seed market in EU is highly concentrated, and the majority of rights are held by several companies (a large proportion of them—German), who can show rather broad plant variety rights and patent portfolios. It seems then that these companies will likely indicate the directions for crop development in the foreseeable future. However, all breeders are currently limited in their choice of breeding methods with regard to the introduction of new products to the EU market. These limitations stem from a practical inability to introduce GM products for cultivation in the EU. Another obstacle is the legal uncertainty regarding the status of the products of NBTs, which may work as a deterring factor in choosing a breeding method, since treating its products as GMOs reguiring authorization effectively renders them unsuitable for the development of new varieties for the EU market. These factors may strongly contribute to the sub-optimal usage of rapeseed’s potential, particularly in comparison to countries with clearer or more liberal policies towards NBTs. This uncertainty and legal obstacles are not encountered by entrepreneurs from other parts of the world (e. g. Argentina or the USA (Eriksson et al., 2019b) and might put European entrepreneurs at a competitive disadvantage.

The analysis of exclusive rights granted for rapeseed-connected inventions and rapeseed varieties shows that there is a growing trend in regard to the numbers of those rights granted. This trend may indicate prospects for the development of the analyzed crop and possibly – its new applications. The growing trend in the number of patent applications can be observed in the case of applications filed with the EPO (counted in thousands), while data from the national patent offices (applications counted in tens or hundreds) show the opposite phenomenon. This fact is rather a symptom of the dwindling popularity of national offices than of the lack of development of rapeseed itself. Trends in the numbers of plant variety rights granted seem concurrent with those of patent applications filed with EPO, showing a significant increase in the last decade. The data on European patents and plant variety rights seem to support the assumed hypothesis that rapeseed has potential to be further developed as a versatile, multi-use crop.

## Author Contributions

EWo and TT drafted the manuscript. EWo conducted the analysis and interpretation of the data related to economic aspects of rapeseed. EWa was responsible for obtaining and interpreting the patent data. TZ and SS presented a chapter on plant variety rights and took part in interpreting patent data. TT studied the conception and design and conducted critical revisions. All the authors reviewed and approved the final manuscript.

## Funding

This work was supported by grants from the National Science Centre, Poland (nos. 2012/06/A/NZ9/00125 and 2014/15/B/NZ9/02312), and by funding from the ERANET-CORNET #22/87/2017 research project named “Innovative processing technology of rapeseed products for poultry nutrition. “ This work was supported by the Swedish Foundation for Strategic Environmental Research (Mistra) through the Mistra Biotech public research program, No. DIA 2013/13. The information and views are those of the authors as individuals and experts in the field, and do not necessarily represent those of the organizations where they work.

## Conflict of Interest

The authors declare that the research was conducted in the absence of any commercial or financial relationships that could be construed as a potential conflict of interest.

## Footnotes

1 The IPC (established by the Strasbourg Agreement, 1971) provides a stratified system of language independent symbols for the classification of patents and utility models in line with the different areas of technology to which they pertain (see World Intellectual Property Organization - WIPO classification).
